# Biofilm Removal from In Vitro Narrow Geometries Using Single and Dual Pulse Er:YAG Laser Photoacoustic Irrigation

**DOI:** 10.3390/microorganisms11082102

**Published:** 2023-08-17

**Authors:** Saša Terlep, Iztok Dogsa, Franja Pajk, David Stopar

**Affiliations:** 1 Fotona d.o.o., Stegne 7, 1000 Ljubljana, Slovenia; sasa.terlep@fotona.com; 2 Department of Microbiology, Biotechnical Faculty, University of Ljubljana, Jamnikarjeva 101, 1000 Ljubljana, Slovenia; iztok.dogsa@bf.uni-lj.si; 3 LA&HA—Laser and Health Academy, Stegne 3, 1000 Ljubljana, Slovenia; franja.pajk@fotona.com

**Keywords:** dental implants, biofilm removal, *Enterococcus faecalis*, narrow geometries, titanium surface, photoacoustic irrigation, Er:YAG-AutoSWEEPS, Er:YAG-SSP, laser induced cavitation, laser peri-implantitis treatment

## Abstract

The disinfection and removal of biofilm from titanium dental implants remains a great challenge in oral medicine. Here we present results of novel photoacoustic irrigation laser modalities for biofilm removal in model geometries mimicking the peri-implant pocket. The efficacy of single pulse (Er:YAG-SSP) and dual pulse (Er:YAG-AutoSWEEPS) photoacoustic irrigation modalities were determined for *Enterococcus faecalis* biofilm decontamination from titanium surfaces in narrow cylindrical and square gap geometries. The density of bacteria as well as the number of live bacteria were determined prior and after different photoacoustic treatments. Both SSP and AutoSWEEPS photoacoustic irrigation techniques removed at least 92% of biofilm bacteria during the 10 s photoacoustic treatment. The effectiveness of cleaning was better in the narrow square gap geometry compared to the cylindrical geometry. The dual pulse Er:YAG-AutoSWEEPS photoacoustic irrigation showed better results compared to SSP modality. No chemical adjuvants were needed to boost the effectiveness of the photoacoustic irrigation in the saline solution. The results imply that photoacoustic irrigation is an efficient cleaning method for debridement and decontamination in narrow geometries and should be considered as a new therapeutic option for the treatment of peri-implant diseases.

## 1. Introduction

Biofilms contribute greatly to chronic infections [1] and their removal remains an unresolved issue. Due to the biofilm role in the etiology of peri-implant diseases the main goal of treatment is in particular to remove biofilms from difficult to reach surfaces [2]. A number of different methods for cleaning and disinfecting titanium dental implants are available, but their effectiveness in removing biofilms is not satisfactory [3,4,5,6,7,8,9,10] and no standard treatment protocol has been proven to be completely effective or superior to the others [8]. Laser-assisted treatments have emerged as useful strategies for resolving peri-implant diseases [11]. Various types of lasers have been in use for the direct (contact) implant irradiation: (neodymium-doped yttrium–aluminum–garnet [Nd:YAG], carbon dioxide [CO_2_], diodes, erbium/chromium-doped yttrium–scandium–gallium–garnet [Er,Cr:YSGG], and erbium-doped yttrium–aluminum–garnet [Er:YAG] [12,13,14]. Due to the high absorption coefficient of Er:YAG in aqueous medium, laser light is efficiently absorbed in a very small volume of irrigant, resulting in photoacoustic cavitation, which activates the flow of the irrigant and brings it to the distal parts of the irrigation system [15,16,17,18,19,20,21]. We showed recently that non-contact photoacoustic fluid streaming and cavitation with Er:YAG-SSP laser modality can effectively remove biofilms from infinite volume geometries [22,23]. Although the results from a large infinite volume geometry are encouraging [15,16,24,25,26,27], it is less well known how biofilms can be removed from confined and difficult to access volumes.

The effect of geometry can be significant and what appears to be a small shift in the system geometry may change the entire flow pattern and therefore cleaning efficiency [17,28]. In narrow geometries the cavitation dynamics is altered because of the friction with the walls and limited space available for rapid fluid movement during the expansion and contraction of the cavitation bubbles. To remedy this super short pulse Er:YAG-SSP modality was specifically developed for the creation of acoustic waves in narrow volume geometries [17,18,29]. However, since SSP does not create shock waves in narrow geometries it was upgraded to SWEEPS (shock wave enhanced emission photoacoustic streaming) modality [17,18], which deliver pulses in pairs where the second pulse puts pressure on the initial bubble and accelerates its collapse. This also generates shock waves in narrow-confined spaces. The optimal time lag between pulses in a pair depends on the volume and geometry of the liquid tank. The correlation is exact; however, its form is only known for defined geometries. A further modification of SWEEPS for geometries where the optimal time lag between the laser pulses is not known was the development of AutoSWEEPS modality. Here the time lag between laser pulses in a pair varies continuously in increments of 10 µs in the range from 200 to 650 µs. This ensures that during each cleaning cycle, there is always a period of an optimal time lag between the pulses required for an efficient emission of shock waves and thus for an optimal flow of irrigant [17,18,28] and better cleaning [19,30,31,32,33,34,35]. In peri-implantology photoacoustic irrigation with SSP and AutoSWEEPS, modalities have not been clinically used.

The objective of this work was to fabricate two different narrow confined geometries and compare the efficiency of two laser pulse modalities for biofilm removal. We hypothesized that Er:YAG-AutoSWEEPS photoacoustic irrigation would better reach inaccessible sites and increase fluid flow rate in narrow geometries which would in turn increase the cleaning of *Enterococcus faecalis* biofilms, the main causative agent of persistent bacterial infection in oral cavity [36,37,38,39]. Biofilms were grown on titanium disks of different dimensions for 3 days to reach maturity. Titanium and its alloy materials are widely used as dental implants, mainly due to high corrosion resistance, good mechanical properties, low toxicity, and bio-inertness [40]. The dental implants are manufactured from pure titanium, cpTi, which is available in four grades numbered 1 to 4, according to the purity and the oxygen content [41,42]. Although titanium grade 4 has the highest mechanical strength titanium grade 2 is often used for dental implants due to lower brittleness, its elasticity and flexibility being beneficial in clinical situations [40,41]. The Er:YAG photoacoustic irrigation was performed in saline solution without the addition of antimicrobial agents. The efficacy of SSP and AutoSWEEPS laser modalities for biofilm removal were tested for 10 and 60 s treatment duration. The density of bacteria as well as the number of live bacteria were determined prior and after different photoacoustic treatments. The results suggest that both SSP and AutoSWEEPS laser modalities are efficient to remove biofilms from confined spaces. In the best-case scenario, 98.5% of biofilm bacteria was removed with AutoSWEEPS modality.

## 2. Materials and Methods

### 2.1. Irrigation Geometries

**Figure 1:**
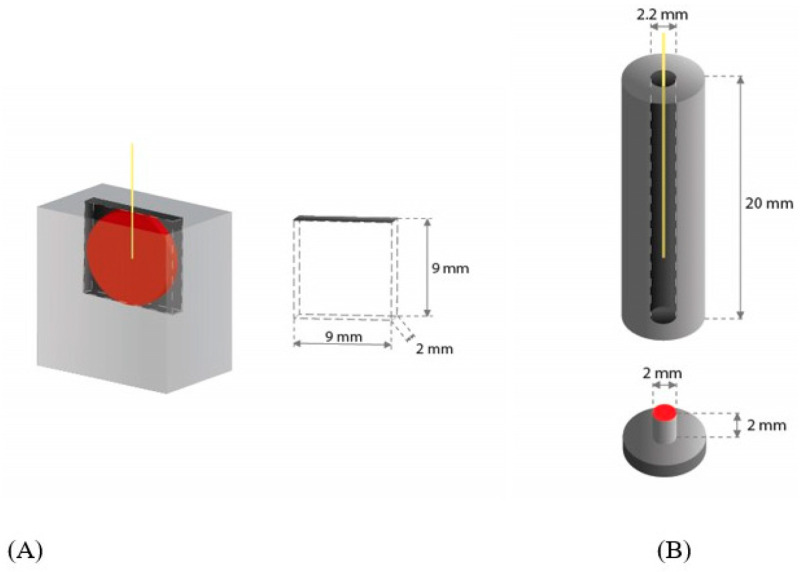
Irrigation models. (**A**) narrow square gap geometry, (**B**) cylindrical geometry. Yellow line indicates the laser tip position, while titanium surfaces covered with biofilms are colored in red.

Two narrow irrigation geometries were fabricated. In the first model system, biofilms were photo-acoustically removed from the narrow square gap geometry mimicking the limited space between the implant biofilm and the gum with the laser fiber tip positioned parallel to the biofilm, while in the cylindrical model, biofilms were removed with the laser tip positioned perpendicular to the biofilm surface. The designs of the two model irrigation systems are depicted in Figure 1.

In the narrow square gap geometry, a box with dimensions 9 mm × 2 mm × 9 mm was produced with milling (polyoxymethylene ErtAcetal, MCAM Tielt, Tielt, Belgium). The titanium disk (7 mm in diameter, 1 mm thick, commercially pure titanium grade 2, VSMPO-AVISMA Corporation, Russia) with in vitro grown biofilm facing the irrigation solution was attached to the box wall with a metal clutch. The biofilm was fully immersed in an irrigation tank filled with saline solution. The laser fiber tip was locked in the gap parallel to the disk at the center of the disc, approximately 0.3 mm from the biofilm surface. The irrigation volume was 123.5 mm^3^.

In the cylindrical irrigation geometry, we used a small titanium cylinder (2 mm in diameter, 2 mm in length). The titanium cylinder with in vitro grown biofilm was placed in a larger cylindrical irrigation tube (polyoxymethylene ErtAcetal, MCAM Tielt) with an inner diameter of 2.2 mm. The biofilm was fully immersed in an irrigation tank filled with saline solution. The laser fiber tip was locked in a position normal to the center of the disc. The vertical distance of the laser tip to the biofilm surface was 5 mm. The irrigation volume was 69.7 mm^3^.

### 2.2. Biofilm Growth

The master stock of *Enterococcus faecalis* Symbioflor was stored at −80 °C. Working stocks of *Enterococcus faecalis* Symbioflor 1 (kindly donated by SymbioGruppe GmbH & CoKG SymbioPharm GmbH) were maintained by weekly subculture on trypticase soy agar (TSA). Before the biofilm experiment, a single colony of *E. faecalis* strain was transferred from TSA and grown overnight in 5 mL trypticase soy broth (TSB) in the dark under aerobic conditions at 37 °C, 200 rpm. Next, sterile titanium disks were placed in a glass Petri dish (d = 15 cm). Titanium disks were pre-prepared by sandblasting for 10 s (FerroEcoBlast Europe; Microblast ceramic beads B120) and subsequent washing with distilled water. The washed disks were dried at room temperature, autoclaved at 134 °C for 20 min, and stored in sterile autoclaving bags prior to use. After each irrigation experiment, the used titanium disks were immersed in 70% ethanol for 60 min, dried at room temperature, sandblasted, washed with distilled water, dried, and autoclaved prior to reuse in the next experiments. The main purpose of sandblasting was to achieve a better retention surface for the attachment of the biofilm as well as to remove any organic material left on the titanium surface that would interfere with the fluorescence measurement. The absence of biofilm and organic material contaminants on the disks after sandblasting was confirmed with confocal laser scanning microscopy. To the sterile titanium disk 1.5 mL (square model) or 2.0 mL (cylindrical model) of overnight *E. faecalis* inoculum (approximately 1 × 10^8^ CFU/mL) was added and then covered with 150 ml (square model) or 200 mL (cylindrical model) of brain heart infusion medium (BHI) and incubated for 72 h in the dark without shaking at 37 °C. The morphology of the growing biofilms was checked with confocal laser scanning microscopy.

### 2.3. Biofilm Photoacoustic Treatments

After the incubation the discs with biofilms were removed from the Petri dish with sterile tweezers and washed thoroughly with 5 mL 0.9% *w*/*v* NaCl (saline solution) using a syringe to remove any loosely attached or suspended bacteria, leaving only the biofilm on the disk. The photoacoustic irrigation was performed with the Fotona laser system LightWalker (Fotona d.o.o) with the following parameters for the super short pulse SSP (50 µs) experiments: wavelength—2940 nm; handpiece—contact H14; fiber tip—FlatSweeps 400/14; energy—20 mJ; frequency—15 Hz; water off; air off. For AutoSWEEPS (2 times ultra short pulse USP: 2 × 25 µs) experiments the parameters were; wavelength: 2940 nm; handpiece—contact H14; fiber tip—FlatSweeps 400/14, energy—2 × 20 mJ; frequency—15 Hz; water off; air off.

For each set of biofilm treatments, a new sterile fibertip was used. Between different samples, fibertip was wiped with propanol mixture (35 g/100 mL 2-propanol and 25 g/100 mL 1-propanol, Incides), splashed with distilled water, and dried.

### 2.4. Determination of Bacterial Viability

After the photoacoustic treatment and prior to the live/dead staining, discs were washed with 5 mL of saline water. We used LIVE/DEAD BacLight bacterial viability kit L7012 (Molecular Probes Inc., Eugene, OR, USA) for microscopy and quantitative analysis. The kit consists of two fluorescent nucleic acid stains, SYTO9 (excitation and emission maxima, 480 and 500 nm, respectively) giving green fluorescence and PI (propidium iodide with excitation and emission maxima, 490 and 635 nm, respectively) which fluoresces in red. The kit was developed to differentiate live and dead bacteria based on cytoplasmic membrane permeability of the two stains. SYTO9 penetrates both viable and nonviable bacteria, while PI penetrates bacteria with damaged plasma membranes only. Consequently, bacterial cells with compromised membranes are red fluorescent and those with intact membranes are green fluorescent [43]. The live/dead stain mixture was prepared according to the producer’s instructions. A mix of 1:1 of both dyes was prepared and diluted in sterile 0.9% *w*/*v* NaCl at a ratio 1.5:500. We used 5 µL of the dye for the square geometry discs and 3 µl of the dye for the cylindrical geometry discs. The disks were incubated in the dark for 5 min at room temperature before microscopy.

### 2.5. Microscopy

After staining, the discs were put on a microscope slide, covered with a protective plastic cap and sealed with wax to protect samples from dehydration. Discs were observed under confocal laser scanning microscope—CLSM (Zeiss Axio Observer Z1 equipped with confocal unit LSM 800, Jena, Germany). On each disc, 2 to 4 randomly selected view fields (127.78 µm × 127.78 µm) across the entire disk surface were acquired by Plan-Apochromat 100×/1.4 NA objective (Zeiss) with up to 15 slices per z-stack. The typical z-step was 1 µm for 100× objective. Alternatively, 2 to 4 randomly selected view fields of the dimensions 319.45 µm × 319.45 µm were acquired by LD Plan-Neofluar 20×/0.4NA objective (Zeiss). The maximal biofilm thickness observed corresponded to ≈15 µm. The images were recorded on two fluorescence channels: a 488 nm laser to acquire green fluorescence (SYTO9 stained cells) and at 561 nm laser to acquire red fluorescence (propidium iodide-stained cells). The pinhole size for the green channel was set to 1.0 AU and the red channel to 1.2 AU. Typically, the frame time was 3.7 s, averaging was set to 4 and 930 × 930-pixel single frame size. The images of the biofilms were analyzed and quantified with Fiji ImageJ 1.52i software, an open-source platform for biological-image analysis, with the use of custom script to avoid personal bias.

### 2.6. Statistical Analysis

We performed 16 independent biological experiments, 9 for the irrigation model A, and 7 for the irrigation model B, with 4–7 discs per treatment group and 2–4 randomly selected scanned areas per disc. All together we made 486 microscopic measurements (164 discs) for the square gap model and 477 measurements (159 discs) for the cylindrical model. The data were linearly transformed to meet the assumptions of a linear mixed model. Analysis was conducted separately for each irrigation model with the treatment group as a fixed factor and the experiment number as a random factor at α = 0.05. Benjamini–Hochberg post hoc analysis was applied to determine the significance of the differences between the control and the treatment groups, between different irrigation durations with the same method, and between SSP and AutoSWEEPS at the same irrigation durations. Statistical analysis was performed in the SPSS program.

## 3. Results

**Figure 2:**
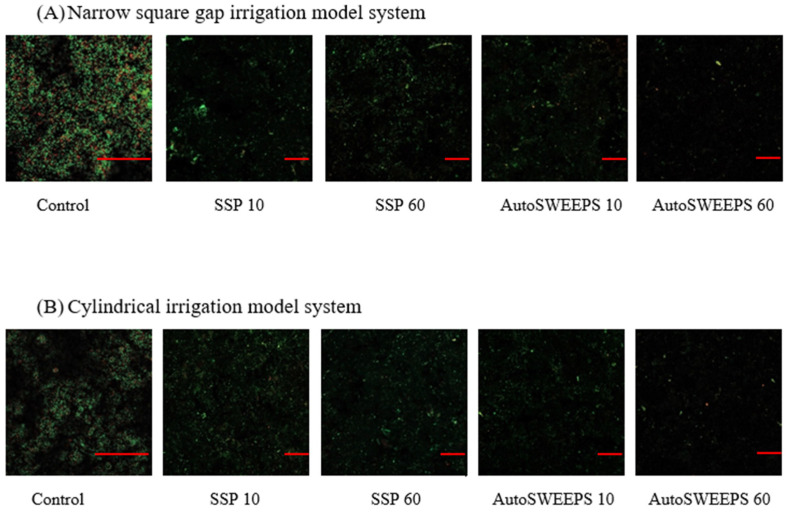
Representative CLSM images of live/dead-stained biofilms of *E. faecalis* on titanium disks treated in (**A**) narrow square gap irrigation model system or (**B**) cylindrical irrigation model system. Shown are control titanium disk without treatment (100× objective) and samples treated with Er:YAG-SSP and Er:YAG-AutoSWEEPS photoacoustic irrigation of 0.9% NaCl (20× objective) for 10 or 60 s. The scale bar on micrographs represents 50 µm.

Figure 2 shows representative micrographs of *E. faecalis* biofilm on titanium discs before treatment and after Er:YAG-SSP and Er:YAG-AutoSWEEPS irrigation in narrow square gap and cylindrical geometries. Qualitatively the results indicate that SSP treatment removed less biofim than AutoSWEEPS treatment. In both photoacoustic modalities increasing the duration of the treatment increased the biofilm removal efficiency.

**Figure 3:**
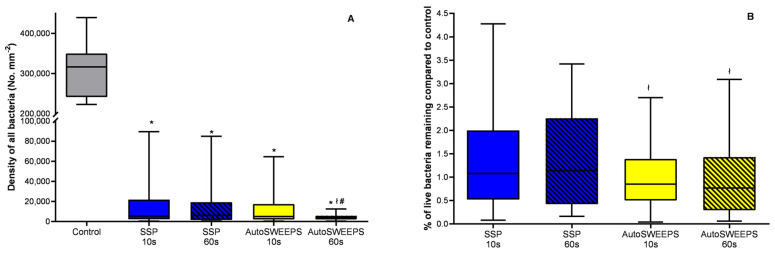
The effectiveness of Er:YAG SSP and AutoSWEEPS modalities for biofilm removal in the narrow square gap model irrigation system. (**A**) The surface density of all bacteria (live + dead) on titanium discs before treatment (control) and after Er:YAG-SSP (blue) or Er:YAG-AutoSWEEPS (yellow) irrigation of saline solution for 10 and 60 s. (**B**) The percentage of live bacteria remaining on titanium discs after Er:YAG-SSP and Er:YAG-AutoSWEEPS irrigation of saline solution for 10 and 60 s compared to the control. * statistically significant difference between the untreated group (control) and the treatment group; ł statistically significant difference between Er:YAG-SSP and Er:YAG-AutoSWEEPS treatments; # statistically significant difference between different time periods for the same laser modality.

To quantify the effectiveness of Er:YAG SSP and AutoSWEEPS modalities on biofilm removal we determined the average bacterial surface density on titanium discs before and after treatments (Figure 3). The SSP modality decreased the number of attached bacteria significantly. There was no significant change between 10 and 60 s of treatment. Compared to SSP treatment the AutoSWEEPS modality gave comparable results after 10 s of treatment. However, the AutoSWEEPS modality was significantly better compared to SSP treatment after 60 s of treatment. The fraction of the viable bacteria that remained on the surface was low (Figure 3B). It is worth noting that the AutoSWEEPS treatment after both 10 and 60 s had a significantly lower fraction of viable bacteria compared to the SSP treatment.

**Figure 4:**
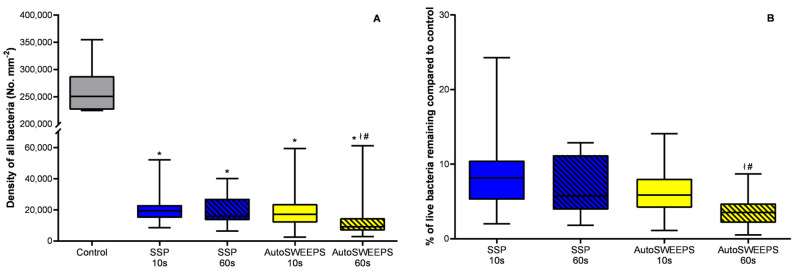
The effectiveness of Er:YAG SSP and AutoSWEEPS modalities for biofilm removal in the cylindrical irrigation model system. (**A**) The surface density of all bacteria (live + dead) on titanium discs before treatment (control) and after Er:YAG-SSP (blue) and Er:YAG-AutoSWEEPS (yellow) irrigation of saline solution for 10 and 60 s. (**B**) The percentage of live bacteria remaining on titanium discs after Er:YAG-SSP and Er:YAG-AutoSWEEPS irrigation of saline solution for 10 and 60 s compared to control. * statistically significant difference between the untreated group (control) and the treatment group; ł statistically significant difference between Er:YAG-SSP and Er:YAG-AutoSWEEPS treatments; # statistically significant difference between different time periods for the same laser modality.

The biofilm removal efficiency in the cylindrical irrigation system is given in Figure 4. Although both laser modalities removed the majority of bacterial cells the numbers of bacteria attached to the surface were larger compared to the square gap geometry. Similar to the square gap geometry the AutoSWEEPS treatment after 60 s was significantly more efficient compared to SSP treatments. The fraction of the viable bacteria that remained on the surface was lowest in the AutoSWEEPS after 60 s of treatment.

## 4. Discussion

Biofilm removal from the peri-implant space with Er:YAG laser had not yet been attempted before this work. The results of this study imply a very good biofilm cleaning efficiency from the model constrained geometries, both with the single pulse SSP and the dual pulse AutoSWEEPS modalities. In narrow square gap geometry up to 95% of the biofilm was removed with the SSP modality and up to 98.5% with the AutoSWEEPS modality. The corresponding cleaning effectiveness values in cylindrical model geometry were lower (93 and 95%, respectively). In both geometries, the AutoSWEEPS modality was more effective while the cleaning effectiveness increased with the duration of the treatment. AutoSWEEPS photoacoustic irrigation methods show good cleaning effectiveness, better than that reported for other decontamination methods [29,44,45]. The dual-pulse SWEEPS was shown previously to be more effective in debris removal compared to the single-pulse SSP modality [46]. Similarly, Yang et al. [29] reported that the dual pulse AutoSWEEPS modality was associated with significantly better removal of debris from the extracted mandibular first and second molars, compared to the single pulse PIPS (photone induced photoacoustic streaming) modality or ultrasonic activated irrigation. Su et al., [47] noticed that the maximum flow speed of the irrigant in the dental root lateral canal (joining mesial root canals containing an isthmus and a single distal canal of first and second molars) was 7 m/s in the single pulse SSP modality and increased to 10 m/s in the SWEEPS modality. Consistently, the results of this study demonstrated that AutoSWEEPS removed more bacteria than SSP in confined geometries. In addition, AutoSWEEPS was more efficient in decreasing the fraction of viable bacteria that remained on the surface after treatments.

The removal of biofilms was more pronounced in square gap geometry compared to cylindrical geometry. The proximity of the laser tip to the biofilm surface in narrow square gap geometry could in addition to cavitation cause biofilm ablation near the laser tip. Although the laser energies (20 mJ) used for the biofilm removal were at or below the estimated threshold for biofilm ablation [48], some direct laser effect on the biofilm cannot be excluded completely. As the microscopy view fields were selected randomly the fact of the absence of the correlation between the biofilm removal efficiency and the distance to the laser tip would argue that direct ablation of the biofilm was not the main mechanism of biofilm removal. Narrow square gap geometry mimics the insertion of the laser tip in the lateral pocket between the gum and the dental implant. In our model system the laser tip was locked in position. Although in clinical practice it would be impossible to lock the position of the laser tip, it is expected that due to the turbulent mixing of the irrigant the cleaning effect could be observed also at large distances from the laser tip and in areas of peri-implant space which could neither be reached directly with mechanical instruments nor with the laser fiber tip.

The geometries which we studied represent only two of many diverse anatomical situations in clinical scenarios [49]. The walls of the model systems were solid which is different from the soft functional tissue of the peri-implant pocket [50,51]. Nevertheless, we envisage that in clinical practice, a solid polymer barrier (i.e., cylindrical or square tube) could be employed to surround the implant for effective surgical cleaning. This will separate the implant from the soft tissue and enable higher efficiency of photoacoustic treatment due to the significant increase in the fluid flow rate facilitated by interaction with the solid walls and the enhanced cleaning action at a distance [30].

The model presented in this study can be improved to better represent clinical situations. We recently demonstrated that cavitation dynamics is significantly affected when cavitation bubbles interact with soft tissue [52]. In the future it would be important to test the effect of the soft walls in contact with the implant on biofilm removal as well as on the removal of multi-species biofilm. A customized mouth tray with the titanium discs exposed to the microbial community in the oral cavity, which would allow the accumulation of a natural biofilm plaque [53], could be tried. Exploring combination therapies and evaluating the long-term effects on implant osseo-integration will further help to guide investigations in the field [54,55,56]. Although the laser energies used in this study were below the ablation threshold, care must be taken not to exceed laser energies of more than 80 mJ/pulse for direct implant treatment, and 50 to 80 mJ/pulse for dental root treatment, as this may cause material damage and possibly delayed growth and adhesion of gingival cells [57].

## 5. Conclusions

The results of this study demonstrate that both the Er:YAG-SSP and Er:YAG-SWEEPS laser modalities have very good cleaning efficiency in the model narrow geometries, better than that reported in the literature. Overall the dual pulse Er:YAG-AutoSWEEPS photoacoustic irrigation modality showed improved results compared to the SSP modality. It is important to note that no chemical adjuvants were needed to boost the effectiveness of the photoacoustic irrigation in the saline solution. The effectiveness of cleaning was better in fabricated narrow square gap geometry compared to cylindrical geometry. The new results support the introduction of both SSP and AutoSWEEPS laser modalities in clinical practice as new tools for the treatment of biofilm related peri-implant diseases.

## Data Availability

The data presented in this study are available on request from the corresponding author.
